# Clinical Color Match and Marginal Discoloration of a Universal Shade Composite Versus a Multi-shade Nanohybrid Composite in Anterior Teeth

**DOI:** 10.7759/cureus.111604

**Published:** 2026-06-27

**Authors:** Esha Fulzele, Neha Jaju, Sneha Rathaur, Pankaj Kumar Gupta, Ankita Singh, Shreyansi Ojha

**Affiliations:** 1 Department of Conservative Dentistry and Endodontics, Rungta College of Dental Sciences and Research, Bhilai, IND

**Keywords:** anterior restorations, color adjustment potential, marginal discoloration, multi-shade composite, universal shade composite

## Abstract

Introduction: Anterior composite restorations require precise color matching and long-term marginal integrity to achieve optimal esthetic outcome. The introduction of structurally colored single‑shade composites such as Omnichroma (Tokuyama Dental Corporation, Tokyo, Japan) has simplified shade selection by utilizing clinical color match (CCM), although their long-term clinical performance relative to conventional multi‑shade composites remains uncertain. The present observational study aimed to compare the CCM and marginal discoloration of a structurally colored universal‑shade composite (Omnichroma) with those of a conventional multi‑shade nanohybrid composite (Beautifil^®^ II*; *Shofu Inc., Kyoto, Japan) in Class III and Class IV anterior restorations over a 12‑month follow‑up.

Materials and methods: This prospective observational clinical study included 100 anterior restorations (50 per material) placed in 50 patients according to the inclusion criteria. Some participants contributed more than one restoration; therefore, the restoration was considered the primary unit of analysis. Restorations were evaluated at baseline and at one, three, six, nine, and 12 months using the modified United States Public Health Service (USPHS) criteria (Alpha, Bravo, Charlie) for restoration appearance (CCM) and marginal integrity (marginal discoloration). Inter‑examiner agreement was assessed using Fleiss’ kappa. Data were analyzed using generalized estimating equations (GEE) and cumulative link mixed models, with significance set at p < 0.05.

Results: For restoration appearance, Omnichroma demonstrated significantly higher clinically acceptable (Alpha+Bravo) rates from six months onward (98% vs. 92% at six months; 94% vs. 80% at 12 months; p < 0.01). The odds ratio for maintaining a clinically acceptable appearance (Charlie) with Beautifil II was 3.42 (95% CI: 1.38-8.47, p = 0.008). For marginal integrity, no statistically significant differences were observed between the materials at any time point, with identical Charlie rates (6%) at 12 months (p > 0.05). Inter‑examiner agreement was substantial to almost perfect (κ = 0.79-0.84).

Conclusion: Within the limitations of this 12‑month observational study, the structurally colored universal‑shade composite (Omnichroma) provided superior CCM compared to the conventional multi‑shade nanohybrid composite (Beautifil II), while both materials exhibited equivalent marginal integrity. Omnichroma simplifies the restorative workflow without compromising the marginal seal, making it a favorable option for anterior Class III and IV restorations.

## Introduction

Resin-based composites have become integral to contemporary restorative dentistry because of their superior esthetics, adhesive potential, and capacity to conserve sound tooth structure [1]. Despite progressive improvements in composite formulations, achieving imperceptible color integration with adjacent tooth structures, particularly in anterior restorations where esthetic demands are highest, remains a persistent clinical challenge [2]. Tooth color is complex and is determined by multiple variables, including anatomical location, age-related alterations, and distinct optical properties of enamel and dentin [3]. Conventional multi-shade composite systems attempt to address this complexity through stratified placement techniques and systematic shade selection; however, these approaches are technique sensitive and largely dependent on clinician experience, which may yield inconsistent outcomes [4,5].

Clinical color match (CCM) is a relevant parameter for characterizing the optical behavior of restorative materials [6]. CCM denotes the ability of a material to blend with the surrounding tooth structure through mechanisms such as light transmission, scattering, and the chameleon effect [7]. These optical phenomena are critical for minimizing the perceptible discrepancies between restorations and natural tooth structures [8]. While CCM influences immediate esthetic integration, long-term clinical performance also depends on resistance to marginal discoloration [9]. Marginal staining compromises esthetics and may indicate deterioration at the tooth-restoration interface; therefore, assessment of both CCM and marginal discoloration is necessary for a comprehensive evaluation of esthetic restorative materials.

Recent material innovations have introduced structurally colored composites, exemplified by Omnichroma (Tokuyama Dental Corporation, Tokyo, Japan), which utilize supra-nano spherical fillers to produce color via light interference rather than traditional pigments [10]. This technology allows a single-shade composite to match a wide range of tooth shades, simplifying shade selection and reducing inventory requirements [11]. Nonetheless, concerns remain regarding their long-term intraoral behavior, particularly with respect to marginal discoloration and color stability [12]. In contrast, conventional multi-shade nanohybrid composites, such as Beautifil II (Shofu Inc., Kyoto, Japan), have demonstrated predictable clinical performance over time, attributable to their established compositions and optical properties [13]. Accordingly, direct clinical comparisons between structurally colored universal-shade composites and established multi-shade systems are warranted.

Although in vitro investigations have reported favorable color-matching characteristics for universal-shade composites, prospective clinical data evaluating their CCM and marginal discoloration in anterior restorations are limited [4,14]. Moreover, long-term intraoral comparative studies between structurally colored universal composites and conventional multi-shade nanohybrid composites remain scarce. The present observational study was therefore undertaken to compare the CCM and marginal discoloration of a structurally colored universal-shade composite (Omnichroma) with those of a conventional multi-shade nanohybrid composite (Beautifil II) in Class III and Class IV anterior restorations over longitudinal follow-up. The primary outcome of this study was the CCM of the two composite systems, operationalized as the proportion of restorations rated clinically acceptable for appearance according to the modified United States Public Health Service (USPHS) criteria [15] over 12 months. The secondary outcome was marginal discoloration, which was assessed as the proportion of restorations with clinically acceptable marginal integrity at each follow-up. The null hypothesis was that there would be no difference between the universal‑shade composite and the multi‑shade nanohybrid composite in terms of clinically acceptable scores for restoration appearance or marginal integrity throughout the 12‑month period. Although several in vitro studies have evaluated the color-matching ability of universal-shade composites, prospective clinical studies directly comparing Omnichroma with Beautifil II in anterior Class III and IV restorations are lacking. Furthermore, evidence regarding their comparative performance in terms of CCM and marginal discoloration during intraoral service remains limited.

## Materials and methods

Study design

This prospective observational comparative clinical study with non-randomized treatment allocation was conducted at the Department of Conservative Dentistry and Endodontics, Rungta College of Dental Sciences and Research, Bhilai, Chhattisgarh, India. The study protocol was approved by the Institutional Ethical Committee of Rungta College of Dental Sciences and Research (reference number: RCDSR/IEC/MDS/2024/P-08). The study protocol adhered to ethical standards and relevant observational study (STROBE) guidelines [16]. Written informed consent was obtained from all participants prior to enrolment.

Sample size determination

The sample size was calculated a priori using G*Power software version 3.1.9.7 (Heinrich Heine University, Düsseldorf, Germany) based on effect estimates (d = 0.59) reported in a previous study [4], using a two-sided alpha level of 0.05, and a statistical power of 80%. The calculated sample size was 45 patients, based on restoration-level comparisons between treatment groups while accounting for a 10% potential loss to follow-up, and a total sample of 50 patients contributing 100 restorations (50 restorations per material group) was considered sufficient.

Participants

The inclusion criteria were as follows: age 20-50 years; presence of at least one anterior tooth requiring esthetic restorations (Ellis class I or II fractures) [17]; absence of clinical or radiographic signs of fistula, pulp exposure, periodontal swelling, abnormal tooth mobility, gingival recession, or alveolar bone loss; and acceptable oral hygiene. The exclusion criteria were periapical pathology (acute apical periodontitis or abscess), heavy tobacco use, deleterious parafunctional habits (e.g., bruxism, traumatic occlusion), and recent dental trauma.

Consecutive participants were recruited from patients attending the Department of Conservative Dentistry and Endodontics between November 2024 and October 2025. All patients who met the eligibility criteria and provided written informed consent during this period were invited to participate in the study. This approach was intended to minimize selection bias and reflect a typical clinical case mix encountered in routine practice. Some patients contributed to more than one eligible restoration, so the unit of analysis for clinical outcomes was the individual restoration rather than the patient. Because multiple restorations could be nested within the same patient, the statistical analysis used methods that accounted for within-subject correlation to avoid inflating type I error.

Clinical procedures

**Figure 1:**
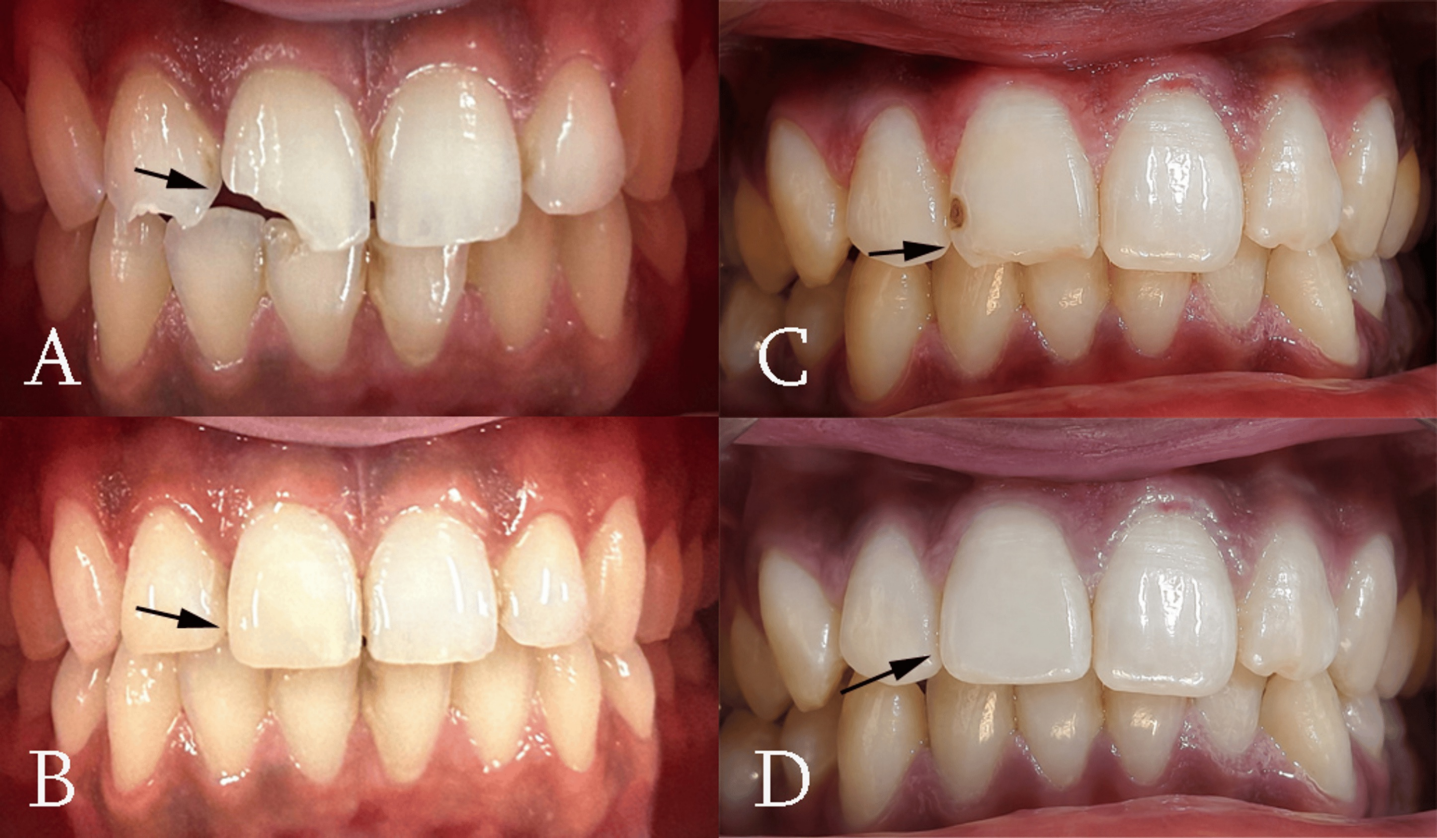
Clinical photographs showing maxillary central incisor restorations (A) preoperative view of a maxillary central incisor with an Ellis fracture (black arrow); (B) postoperative view after restoration with universal-shade composite (Omnichroma; Tokuyama Dental Corporation, Tokyo, Japan)  (black arrow); (C) preoperative view of a maxillary central incisor with Class IV caries  (black arrow); and (D) postoperative view after restoration with conventional multi-shade nanohybrid composite (Beautifil II; Shofu Inc., Kyoto, Japan) (black arrow). Original patient image from study used with consent.

Eligible patients underwent esthetic anterior restorations with either a structurally colored universal‑shade composite or a conventional multi‑shade nanohybrid composite after explanation of the available options. Consequently, the two material groups represent naturally formed, clinically comparable cohorts rather than formally randomized arms. The baseline characteristics of the restorations were recorded to verify the comparability between the groups. The treatment allocation reflected routine clinical practice while ensuring inter-subject comparison; each participant received anterior restorations, either a single-shade universal composite (Figure 1A, 1B) or a multi-shade nanohybrid composite (Figure 1C, 1D). All procedures were performed by a single experienced operator following standardized clinical protocols to minimize procedural variability.

After rubber dam isolation, all carious tissue and unsupported enamel were removed using a #330 carbide bur (Prima Dental, Quedgeley, Gloucestershire, United Kingdom) with a water coolant. For fractured anterior teeth requiring Class IV restorations, a labial enamel bevel was prepared to enhance the esthetic integration. Selective enamel etching was performed with 37% phosphoric acid (Rizhao HuGe Biomaterials Company, Ltd., Rizhao, Shandong, China) according to the manufacturer’s recommendations. A bonding agent (Fusion Bond 5; Prevest DenPro Limited, Jammu, Jammu & Kashmir, India) was then applied and light-cured, following the instructions specific to each adhesive system. For the conventional multi-shade nanohybrid composite (Beautifil II), an appropriate shade was selected based on the adjacent tooth structure (shade selection was performed prior to rubber dam isolation using the manufacturer's shade guide under standardized daylight-equivalent illumination.

Teeth were cleaned with pumice, maintained in a hydrated state, and shade matching was completed before enamel dehydration occurred; for the universal‑shade composite (Omnichroma), no shade selection was necessary owing to its structural color-matching technology. The composite was placed incrementally in oblique layers not exceeding 2 mm in thickness, and each increment was light-cured for 20 s using a calibrated LED light-curing unit (Bluephase N; Ivoclar Vivadent AG, Schaan, Liechtenstein) with a wavelength range of 385-515 nm. The operators strictly followed each manufacturer's recommended handling and placement techniques for the respective materials. After complete curing, restorations were finished and polished using a standardized polishing protocol and composite finishing and polishing kit (Astropol; Ivoclar Vivadent AG) to obtain a smooth, glossy surface outcome assessment.

The primary outcomes were CCM and marginal discoloration. Clinical evaluations were performed at baseline (immediately after restoration) and one, three, six, nine, and 12 months after restoration. Two calibrated examiners independently assessed restorations under standardized daylight-equivalent lighting. The examiners were blinded to the study hypothesis and the clinical records of the patients during evaluation; however, blinding of the specific composite material was not feasible because the appearance of the materials could sometimes be recognized clinically. To minimize potential detection bias, examiners were calibrated before the study using a set of training restorations and agreed on the operational definitions of Alpha, Bravo, and Charlie scores for each USPHS criterion. Restoration appearance and marginal integrity were scored using the USPHS criteria: Alpha (ideal), Bravo (clinically acceptable with minor deviation), or Charlie (clinically unacceptable) [15]. Inter-examiner agreement was assessed prior to the study and was monitored periodically. Although CCM is frequently evaluated using instrumental techniques such as spectrophotometry and ΔE calculations, the present study employed modified USPHS appearance criteria because they assess clinically perceived color integration and esthetic acceptability under routine clinical conditions. Therefore, restoration appearance was used as a clinical surrogate for color adjustment performance.

Data management and statistical analysis

Clinical findings were recorded on standardized case report forms and entered into Microsoft Excel (Microsoft Corporation, Redmond, Washington, United States). Descriptive statistics summarized participant demographics and baseline characteristics. Statistical analyses were performed using IBM SPSS Statistics for Windows, version 23 (IBM Corp., Armonk, New York, United States). Inter-examiner agreement was assessed using Fleiss’ kappa. Baseline characteristics were compared using the chi-square and independent t-tests. Because repeated measurements were obtained from the same restorations over time, generalized estimating equations (GEE) with an exchangeable correlation structure and logit link function were used to compare the proportions of clinically acceptable (Alpha + Bravo) scores between materials across follow-up visits. This approach accounts for within-patient and within-patient correlations over time, and provides robust standard errors in the presence of incomplete follow-up. Odds ratios (ORs) with 95% confidence intervals (CIs) were calculated to quantify the relative likelihood of clinically acceptable outcomes for the universal‑shade composite compared with the multi‑shade composites. All analyses were performed at a significance level of 0.05.

## Results

Table 1 presents the Fleiss’ kappa values for inter-examiner agreement on the USPHS criteria at pre-study, six months, and 12 months. For restoration appearance, agreement was almost perfect pre-study (κ = 0.82, p < 0.001), remained substantial at six months (κ = 0.79, p < 0.001), and returned to almost perfect at 12 months (κ = 0.81, p < 0.001). For marginal integrity, pre-study agreement was κ = 0.84 (p < 0.001), with similarly high values at six months (κ = 0.80, p < 0.001) and 12 months (κ = 0.82, p < 0.001). All kappa values exceeded 0.79, indicating substantial to almost perfect agreement. These results confirm the excellent reproducibility of USPHS scoring across examiners throughout the study.

**Table 1:** Inter-examiner agreement (Fleiss’ Kappa) for USPHS criteria. Kappa (κ) values indicate inter-rater agreement; CI = confidence interval; Z-value and p-value were calculated to assess the statistical significance of agreement. *p < 0.001 = significant. USPHS: United States Public Health Service

Criterion	Time Point	Kappa (κ)	95% CI	Z‑value	p‑value
Restoration appearance (clinical color match)	Pre‑study	0.82	0.76–0.88	12.3	<0.001*
	6 months	0.79	0.73–0.85	11.9	<0.001*
	12 months	0.81	0.75–0.87	12.1	<0.001*
Marginal integrity (discoloration)	Pre‑study	0.84	0.78–0.90	13.0	<0.001*
	6 months	0.80	0.74–0.86	12.0	<0.001*
	12 months	0.82	0.76–0.88	12.4	<0.001*

Table 2 summarizes the baseline characteristics of Omnichroma (n=50) and Beautifil II (n=50). No significant differences were observed between the groups. For the restoration class, Omnichroma had 28 (56%) Class III and 22 (44%) Class IV, and Beautifil II had 30 (60%) Class III and 20 (40%) Class IV. The tooth type distribution showed incisors in 41 (82%) and 38 (76%), and canines in nine (18%) and 12 (24%), respectively. Patient age (32.5 ± 9.4 vs. 33.1 ± 10.2 years) and sex distribution were also comparable. These findings confirmed that the groups were comparable at the baseline.

**Table 2:** Baseline characteristics of restorations SD = standard deviation; χ² = chi-square test; t = Student’s t-test; p-values refer to comparisons between the Omnichroma and Beautifil II groups. The difference was considered statistically significant at p < 0.05.

Characteristic		Omnichroma (n= 50), n (%)	Beautifil II (n= 50), n (%)	Test statistic	p‑value
Restoration class, n (%)	Class III	28 (56%)	30 (60%)	χ² = 0.16	0.689
	Class IV	22 (44%)	20 (40%)		
Tooth type (incisor/canine), n (%)	Incisor	41 (82%)	38 (76%)	χ² = 0.37	0.543
	Canine	9 (18%)	12 (24%)		
Patient age (mean ± SD)	In years	32.5 ± 9.4	33.1 ± 10.2	t = –0.30	0.764
Patient sex, n (%)	Male	22 (44%)	24 (48%)	χ² = 0.16	0.689
	Female	28 (56%)	26 (52%)		

Table 3 shows the USPHS scores for the appearance of restoration after 12 months. In the Omnichroma group, ideal (Alpha) scores decreased gradually from 50 (100%) at baseline to 39 (78%) at 12 months, while Bravo (acceptable) increased to 8 (16%), and Charlie (unacceptable) appeared in only three (6%) by 12 months. In contrast, Beautifil II showed more rapid deterioration: Alpha scores dropped from 50 (100%) at baseline to 23 (46%) at 12 months, with Bravo rising to 17 (34%) and Charlie reaching 10 (20%) at the final follow-up. Notably, no Charlie scores were recorded for either group at baseline or at one month. At 12 months, the proportion of clinically unacceptable restorations was substantially lower for Omnichroma (3/50, 6%) than for Beautifil II (10/50, 20%). These data suggest superior CCM for structurally colored composites.

**Table 3:** Restoration appearance (clinical color match) – distribution of USPHS scores. Alpha indicates ideal clinical performance, Bravo indicates acceptable clinical performance, and Charlie indicates unacceptable clinical performance. USPHS: United States Public Health Service

Material	Time point	Alpha (ideal), n (%)	Bravo (acceptable), n (%)	Charlie (unacceptable), n (%)
Omnichroma	Baseline	50 (100%)	0 (0%)	0 (0%)
	1 month	48 (96%)	2 (4%)	0 (0%)
	3 months	46 (92%)	4 (8%)	0 (0%)
	6 months	44 (88%)	5 (10%)	1 (2%)
	9 months	41 (82%)	7 (14%)	2 (4%)
	12 months	39 (78%)	8 (16%)	3 (6%)
Beautifil II	Baseline	50 (100%)	0 (0%)	0 (0%)
	1 month	44 (88%)	6 (12%)	0 (0%)
	3 months	38 (76%)	11 (22%)	1 (2%)
	6 months	32 (64%)	14 (28%)	4 (8%)
	9 months	27 (54%)	16 (32%)	7 (14%)
	12 months	23 (46%)	17 (34%)	10 (20%)

Table 4 presents USPHS scores for marginal integrity. Both groups started with 50 (100%) Alpha scores at baseline. At one month, Omnichroma had 49 (98%) Alpha and one (2%) Bravo; Beautifil II had 48 (96%) Alpha and two (4%) Bravo. Over time, deterioration was gradual and nearly identical between materials. At 12 months, Omnichroma showed 39 (78%) Alpha, eight (16%) Bravo, and three (6%) Charlie; Beautifil II showed 37 (74%) Alpha, 10 (20%) Bravo, and three (6%) Charlie. The cumulative Charlie rates were identical (3/50, 6% in each group) at final follow‑up. No significant differences were observed at any time point. These results indicate that marginal discoloration progresses similarly for both composite types, with the structurally colored material performing equally well as the conventional nanohybrid composite in terms of marginal integrity.

**Table 4:** Marginal integrity (marginal discoloration) – distribution of USPHS scores. Alpha indicates ideal clinical performance, Bravo indicates acceptable clinical performance, and Charlie indicates unacceptable clinical performance. USPHS: United States Public Health Service

Material	Time point	Alpha (ideal), n (%)	Bravo (acceptable), n (%)	Charlie (unacceptable), n (%)
Omnichroma	Baseline	50 (100%)	0(0%)	0(0%)
	1 month	49 (98%)	1 (2%)	0(0%)
	3 months	47 (94%)	3 (6%)	0(0%)
	6 months	45 (90%)	4 (8%)	1 (2%)
	9 months	42 (84%)	6 (12%)	2 (4%)
	12 months	39 (78%)	8 (16%)	3 (6%)
Beautifil II	Baseline	50 (100%)	0(0%)	0(0%)
	1 month	48 (96%)	2 (4%)	0(0%)
	3 months	46 (92%)	4 (8%)	0(0%)
	6 months	43 (86%)	6 (12%)	1 (2%)
	9 months	40 (80%)	8 (16%)	2 (4%)
	12 months	37 (74%)	10 (20%)	3 (6%)

Table 5 compares “clinically acceptable” (Alpha+Bravo) rates between materials using GEE. For restoration appearance, the acceptable rates were 100% in both groups at one and three months. At six months, Omnichroma (98%) was superior to Beautifil II (92%), with an = 3.12 (p = 0.004). This advantage increased at nine (96% vs. 86%, OR = 4.10, p < 0.001) and 12 months (94% vs. 80%, OR = 4.89, p < 0.001). For marginal integrity, acceptable rates were 100% in both groups at one and three months and equal at six months (98% vs. 98%, OR = 1.00, p = 1.000), nine months (96% vs. 96%, p = 1.000), and 12 months (94% vs. 94%, p = 1.000). Thus, Omnichroma significantly outperformed Beautifil II in color adjustment from six months onward, with no difference in marginal discoloration.

**Table 5:** Comparison of “clinically acceptable” (Alpha + Bravo) rates – GEE analysis. Values are presented as percentages; OR = odds ratio; CI = confidence interval; χ² (Wald) = Wald chi-square test statistic. A dash indicates that the odds ratio was not estimated because there was no difference between groups or the outcome was identical in both groups. *p < 0.05. GEE: generalized estimating equations.

Criterion	Time	Omnichroma	Beautifil II	Odds Ratio (OR)	95% CI	χ² (Wald)	p‑value
Restoration appearance (clinical color match)	1 month	100%	100%	-	-	-	-
	3 months	100%	98%	2.04	0.97–4.29	3.58	0.058
	6 months	98%	92%	3.12	1.42–6.85	8.17	0.004*
	9 months	96%	86%	4.10	1.89–8.91	12.80	<0.001*
	12 months	94%	80%	4.89	2.31–10.35	18.15	<0.001*
Marginal integrity (discoloration)	1 month	100%	100%	-	-	-	-
	3 months	100%	100%	-	-	-	-
	6 months	98%	98%	1.00	0.45–2.22	0.00	1.000
	9 months	96%	96%	1.00	0.48–2.10	0.00	1.000
	12 months	94%	94%	1.00	0.51–1.96	0.00	1.000

## Discussion

This prospective observational study compared the clinical performance of a structurally colored universal‑shade composite and a conventional multi‑shade nanohybrid composite in anterior Class III and IV restorations over 12 months. The universal‑shade composite exhibited higher clinically acceptable rates for restoration appearance from six months onward, with fewer restorations rated clinically unacceptable for color matching at 12 months. In contrast, both materials showed comparable marginal integrity throughout the follow-up period with similar frequencies of marginal discoloration and clinically unacceptable scores.

Clinical color match

The present study demonstrated that Omnichroma (structurally colored universal‑shade composite) exhibited significantly superior CCM than Beautifil II (conventional multi‑shade nanohybrid composite) from six months onward, with higher clinically acceptable rates (94% vs. 80% at 12 months) and fewer Charlie scores (6% vs. 20%). This superiority can be explained by the fundamental differences in their optical designs. Omnichroma utilizes supraspherical fillers (approximately 260 nm) that generate structural colors via light interference rather than traditional pigments [5,8]. This technology produces a broad spectral reflection that adapts to the surrounding tooth structure, enhancing the “chameleon effect” and allowing a single shade to match various tooth shades [18]. In contrast, Beautifil II is a Giomer‑based multi‑shade composite that relies on conventional pigmentation and requires precise shade selection; therefore, its CCM is inherently limited by the accuracy of the chosen shade and the clinician’s skill [19].

Several studies have supported the favorable CCM of single-shade composites. Islam et al. reported that Omnichroma demonstrated a clinically acceptable blending effect when placed against different shades of conventional resin composites in vitro [4]. Sanchez et al. confirmed that instrumental and visual evaluations both detect superior color blending in structurally colored materials [3]. Baghizadeh et al. specifically assessed Omnichroma in posterior restorations and found acceptable shade matching, further supporting its universal applicability [7]. Rosa et al. demonstrated that single-shade composites provide effective color matching and even color recovery in large composite restorations [9].

However, there is conflicting evidence regarding this. In a randomized controlled trial by Anwar et al., a multi‑shade composite group showed a statistically significantly higher prevalence of ideal (Alpha) color match scores compared to a single‑shade universal composite at nine and 12 months, albeit in posterior restorations [2]. This discrepancy may be attributed to the fact that anterior restorations (present study) have different optical demands and are influenced more by the chameleon effect, whereas posterior restorations may be less forgiving. Additionally, Zulekha et al. found comparable clinical performance between one-shade universal and nanohybrid composites in primary maxillary incisors, suggesting that material selection may be tooth- and site-dependent [13]. Thus, while Omnichroma excels in CCM for anterior Class III/IV restorations, its advantage may vary according to the clinical scenario.

Marginal integrity (discoloration)

Both Omnichroma and Beautifil II exhibited equivalent marginal integrity throughout the 12‑month follow‑up, with identical Charlie rates (6% each) and no statistically significant differences in the clinically acceptable rates at any time point. This finding indicates that the simplified placement protocol and unique filler technology of Omnichroma do not compromise the marginal seal compared to a well-established nanohybrid Giomer.

The marginal seal of resin composites depends on several factors, including polymerization shrinkage, adhesion to tooth structure, filler morphology, and resistance to hydrolytic degradation. Beautifil II, as a Giomer, releases and recharges fluoride, which may theoretically reduce secondary caries and marginal staining [19]. However, in the present study, this did not translate to superior marginal integrity. Omnichroma supraspherical fillers (260 nm) are uniformly dispersed and may reduce polymerization stress by facilitating more homogeneous light transmission and curing [5,8]. Moreover, the absence of conventional pigment particles may influence optical stability [20]; however, the present study did not directly evaluate water sorption or staining mechanisms, and further investigation is warranted.

The existing literature on marginal discoloration in single-shade composites is limited but evolving. Anwar et al. reported no significant difference in marginal discoloration between single‑shade and multi‑shade composites at 12 months, consistent with our results [2]. In a 12‑month clinical evaluation of composite resins in Class III restorations, Loguercio et al. emphasized that marginal integrity was more dependent on the adhesive technique and operator skill than on the composite material itself [21]. In an in vitro dye leakage study, Bajabaa et al. found that different resin composites (including nanohybrids) showed variable microleakage, but no single composition consistently outperformed the others [1]. This supports our observation that the marginal seal is largely technique‑sensitive rather than material‑specific.

A potential concern with structurally colored composites is their high filler load and spherical filler geometry, which might affect their rheological properties and adaptation to cavity walls [14,21]. However, the present study’s equivalent marginal integrity outcomes suggest that when placed using a standardized incremental technique and a fifth-generation bonding agent, Omnichroma achieves satisfactory marginal adaptation. Future long-term studies beyond 12 months are needed to determine whether a similar marginal seal persists over extended intraoral exposure to thermal and mechanical stresses.

Several limitations of this study should be acknowledged when interpreting these findings. First, the treatment allocation was observational and reflected routine clinical decision-making rather than randomized assignment, which may have introduced selection bias despite the comparable baseline characteristics of the two groups. Second, although examiners were calibrated and blinded to the study hypothesis, they could not be fully blinded to the restorative material, raising the possibility of detection bias in the subjective USPHS scoring. Because CCM was evaluated using clinical appearance scores rather than instrumental color measurements, the results reflect perceived clinical color matching rather than objective optical color differences. Third, the study was conducted at a single academic center with all procedures performed by a single experienced operator, which enhances procedural standardization but may limit the generalizability of the results to other settings and levels of operator experience. Finally, the 12‑month follow‑up period is relatively short for esthetic restorative outcomes, and longer-term evaluations are needed to determine whether the observed differences in CCM persist or change over time. 

Clinical implication

Within these constraints, the present findings suggest that structurally colored universal‑shade composites can simplify the restorative workflow in anterior Class III and IV cavities by reducing the need for shade selection while maintaining, and in terms of appearance, potentially improving short-term esthetic outcomes relative to a multi‑shade nanohybrid composite. However, clinicians should consider individual tooth characteristics, extreme shade situations, and patient-specific esthetic expectations when selecting materials, and should not regard universal‑shade systems as a complete replacement for multi‑shade composites in all clinical scenarios.

## Conclusions

Within the limitations of this single-center, 12‑month prospective observational study, the structurally colored universal‑shade composite demonstrated superior CCM compared to the conventional multi‑shade nanohybrid composite in anterior Class III and IV restorations, while both materials exhibited similar marginal integrity. These results support the use of universal‑shade composites as a viable option for anterior esthetic restorations, particularly when the simplification of shade selection is desired. Further randomized clinical trials with longer follow-up periods and diverse clinical settings are required to confirm and extend these findings.
